# Using elastography-based multilayer perceptron model to evaluate renal fibrosis in chronic kidney disease

**DOI:** 10.1080/0886022X.2023.2202755

**Published:** 2023-04-19

**Authors:** Ziman Chen, Tin Cheung Ying, Jiaxin Chen, Chaoqun Wu, Liujun Li, Hui Chen, Ting Xiao, Yongquan Huang, Xuehua Chen, Jun Jiang, Yingli Wang, Wuzhu Lu, Zhongzhen Su

**Affiliations:** a Department of Health Technology and Informatics, Hong Kong Polytechnic University, Kowloon, Hong Kong; b Department of Ultrasound, Fifth Affiliated Hospital of Sun Yat-sen University, Zhuhai, P.R. China; c Central Lab, Liver Disease Research Center, The Affiliated Hospital of Yunnan University, Kunming City, Yunnan Province, P.R. China; d Department of Radiology, The Second People’s Hospital of Shenzhen, Shenzhen, P.R. China; e Ultrasound Department, EDAN Instruments, Inc, Shenzhen, P.R. China

**Keywords:** Chronic kidney disease, renal fibrosis, shear wave elastography, multilayer perceptron, machine learning

## Abstract

**Background:**

Given its progressive deterioration in the clinical course, noninvasive assessment and risk stratification for the severity of renal fibrosis in chronic kidney disease (CKD) are required. We aimed to develop and validate an end-to-end multilayer perceptron (MLP) model for assessing renal fibrosis in CKD patients based on real-time two-dimensional shear wave elastography (2D-SWE) and clinical variables.

**Methods:**

From April 2019 to December 2021, a total of 162 patients with CKD who underwent a kidney biopsy and 2D-SWE examination were included in this single-center, cross-sectional, and prospective clinical study. 2D-SWE was performed to measure the right renal cortex stiffness, and the corresponding elastic values were recorded. Patients were categorized into two groups according to their histopathological results: mild and moderate-severe renal fibrosis. The patients were randomly divided into a training cohort (*n* = 114) or a test cohort (*n* = 48). The MLP classifier using a machine learning algorithm was used to construct a diagnostic model incorporating elastic values with clinical features. Discrimination, calibration, and clinical utility were used to appraise the performance of the established MLP model in the training and test sets, respectively.

**Results:**

The developed MLP model demonstrated good calibration and discrimination in both the training [area under the receiver operating characteristic curve (AUC) = 0.93; 95% confidence interval (CI) = 0.88 to 0.98] and test cohorts [AUC = 0.86; 95% CI = 0.75 to 0.97]. A decision curve analysis and a clinical impact curve also showed that the MLP model had a positive clinical impact and relatively few negative effects.

**Conclusions:**

The proposed MLP model exhibited the satisfactory performance in identifying the individualized risk of moderate-severe renal fibrosis in patients with CKD, which is potentially helpful for clinical management and treatment decision-making.

## Introduction

Chronic kidney disease (CKD) is one of the leading non-communicable diseases worldwide. Globally, 1.2 million deaths from CKD were recorded in 2017, with a worldwide prevalence of 9.1% (697.5 million cases) [1]. The high morbidity and mortality of CKD pose a serious threat to both the physiological and mental health of patients, and impose an unprecedented burden on national healthcare systems [2,3]. CKD is a progressive form of kidney impairment accompanied by structural and functional damage [4]. Irrespective of the underlying etiology, renal fibrosis is the prominent pathological feature of CKD and the pathway leading to end-stage renal disease [5]. Indeed, renal fibrosis has been identified as one of the most independent risk factors for CKD progression and poor prognosis [6–8]. Therefore, an accurate diagnosis of the stages of renal fibrosis is of vital importance with respect to clinical treatment and patient prognosis.

Renal biopsy is still the gold standard for detecting and staging renal fibrosis [9]. However, it is difficult to perform biopsies repeatedly to monitor disease development and perform longitudinal follow-up since renal biopsy is an invasive procedure [10, 11]. As a leading-edge modality in the field of medical ultrasound (US) imaging, real-time two-dimensional shear wave elastography (2D-SWE) is applied to detect target tissue elastic value and reflect the mechanical property noninvasively [12]. It has demonstrated good promise in judging the condition, determining the treatment regimen, and evaluating the therapeutic response [13, 14]. Currently, some applications of 2D-SWE in the field of diagnosis and staging renal fibrosis in patients with CKD have been reported, but the diagnostic accuracy, sensitivity, and specificity remain limited [15, 16].

Machine learning, as a state-of-the-art analysis and modeling technique, has developed dramatically in recent years and emerged as a powerful tool in the medical domain [17]. The multilayer perceptron (MLP) classifier, a machine learning method based on a feed-forward artificial neural network model, is important in nonlinear fitting analysis because it has high fault tolerance and self-adaptability [18]. Previous studies have applied the MLP approach to prediction model construction by integrating clinical features with imaging characteristics and achieved outstanding results [19–21]. However, to the best of our knowledge, no study has yet explored whether incorporating 2D-SWE into clinical features by using the MLP approach could be used for the assessment of renal fibrosis in CKD patients. Therefore, in this study, we aimed to develop a diagnostic MLP model based on 2D-SWE and easily accessible clinical features to assess renal fibrosis in patients with CKD and further validate its practical performance as well as utility.

## Patients and methods

This was a single-center, cross-sectional, and prospective clinical study. The study protocol was reviewed and approved by our institution’s Ethics Committee before the commencement of the study. All participants gave their written informed consent.

### Study cohort

During the period April 2019 to December 2021, patients with CKD who underwent a 2D-SWE examination and a renal biopsy at our institution were prospectively and consecutively included. The criteria for study enrollment were as follows: (1) a diagnosis of CKD was made in accordance with the Kidney Disease Improving Global Outcomes (KDIGO) guidelines (2012) [22]; (2) a 2D-SWE examination was routinely conducted on each participant before renal biopsy; (3) an assessment of renal fibrosis by means of a kidney biopsy specimen was performed in all cases, and (4) a complete medical history and laboratory investigations were obtained from all subjects. The exclusion criteria were the following: (1) cases had multiple renal cysts, nephrolithiasis, hydronephrosis, or renal masses that could interfere with 2D-SWE examination; (2) cases failed to control breathing as directed during 2D-SWE procedure; (3) cases could not be appropriately evaluated by 2D-SWE procedure due to technical reasons (e.g., obesity, mental tension); and (4) the quality of the renal biopsy tissue was insufficient (less than 10 mm in length or fewer than 10 glomeruli). According to eligibility criteria, 162 patients were finally included in this study. Following this, the entire cohort was randomly split into two datasets as per a 7:3 ratio: a training cohort (*n* = 114) and a test cohort (*n* = 48). The training set was used for model construction, while the test set was used for independent model evaluation. It is undeniable that large-scale datasets facilitate the training and testing of models in the field of medical data analysis. However, it is worth noting that the availability of large-scale datasets is not a universal phenomenon, particularly in the context of some prospective clinical studies that require pathological evidence. In such cases where data volume is limited, it becomes necessary to strike a delicate balance between the sample size necessary for modeling and the sample size requisite for confirming the model’s capability. The utilization of a test set that constitutes a limited proportion of the total data is a commonly accepted practice [23]. This approach is motivated by two primary considerations. First, it is acknowledged that, although the test set may comprise a relatively small fraction of the overall data, it is capable of providing sufficient cases to evaluate the model’s generalization. Second, if the test set were to be of substantial size, it would result in an inefficient utilization of the data. A widely adopted strategy in such scenarios involves the application of a 7:3 ratio for these purposes [24–26]. It is critical to emphasize that, even when dealing with small-scale clinical studies, it is crucial to ensure that the data partitioned into the training and test sets is representative of the population of interest and derived from the same distribution [27]. Additionally, the data must be randomly assigned to the training and test sets in order to mitigate any potential biases in the evaluation of the model.

### Clinical feature

Demographic information (including age, sex, and body mass index), liquid biopsy indicators (including blood urea nitrogen, serum albumin, serum uric acid, serum creatinine, urinary albumin creatinine ratio (UACR), and estimated glomerular filtration rate (eGFR)), and comorbidity (e.g., cardiovascular disease, diabetes, and hypertension) were obtained from each participant. The eGFR was calculated using the CKD epidemiology collaboration (CKD-EPI) formula [28]. The CKD-EPI formula is more accurate than the Modification of Diet in Renal Disease (MDRD) formula for determining eGFR, as recommended by the KDIGO guideline (2012), especially for values greater than 60 mL/min/1.73 m^2^ [22]. Furthermore, the CKD-EPI equation is preferred in general practice and public health [29]. As for the Cockcroft-Gault equation, it overestimates renal function, and the estimation of GFR is less accurate [30]. Liquid biopsy indicators were collected according to laboratory standard operating procedures within a week prior to the renal biopsy. Diabetes and hypertension were identified based on physician diagnosis with International Classification of Diseases (ICD) codes or documentation of patients taking insulin, oral hypoglycemic agents, and anti-hypertensive drugs. Cardiovascular disease was defined as the presence of heart failure, coronary heart disease, stroke, or peripheral vascular disease.

### 2D-SWE examination

**Figure 1.**
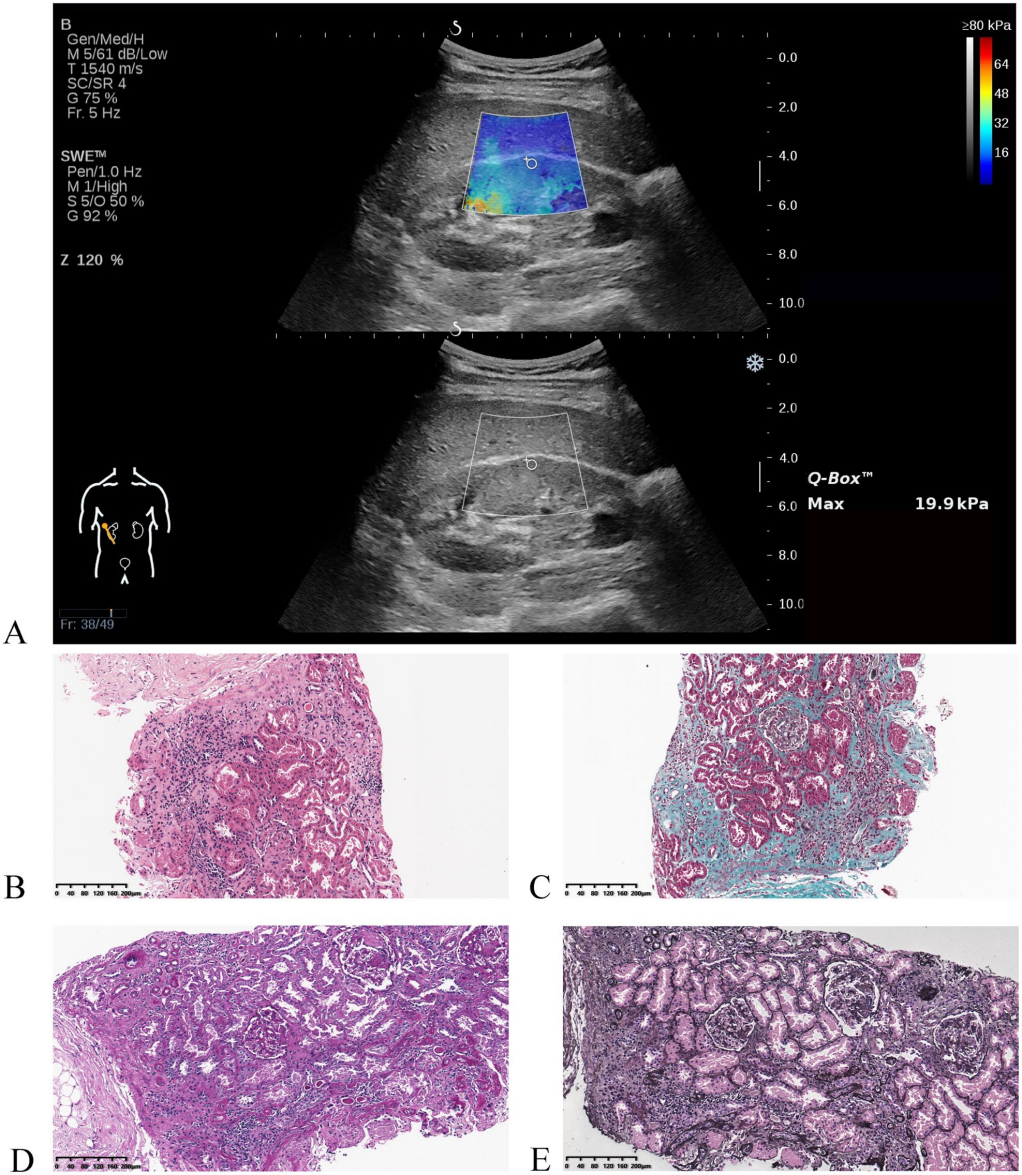
A representative example of shear wave elastography imaging (A) and histopathological analysis using various stainings (B-E). (A) A color-coded shear wave elastogram and a corresponding conventional ultrasound image of a patient with chronic kidney disease. Images of a 10× objective kidney biopsy stained with (B) hematoxylin-eosin stain, (C) Masson’s trichrome stain, (D) periodic acid-Schiff stain, and (E) methenamine silver stain taken from this patient.

Within two days prior to the renal biopsy procedure, a board-certified radiologist with over three years’ experience in abdominal elastography conducted a 2D-SWE examination on the patients. The radiologist was blinded to the information regarding the clinical and laboratory status of the patient. A 2D-SWE examination was conducted when patients lay supine on an US examination table using an Aixplorer US imaging system (Supersonic Imagine, Aixen-Provence, France) equipped with a SC6-1 broad band convex array probe (frequency: 1–6 MHz). In the 2D-SWE examination, participants were required to empty their bladders completely before the procedure and also hold their breath for a few seconds during the procedure. Measurements were performed in the right renal coronal plane, with the transducer parallel to the renal-axis view and without applying any physical pressure to the patient. In conventional US, the renal length (from the upper pole to the lower pole), renal parenchymal thickness (from the outer renal margin to the outer margin of the renal sinus), and renal inter-lobar artery resistive index (RI) were measured. A trapezium-shaped, color-coded elasticity image box measuring 4 × 3 cm was placed in the mid-region of the kidney. Then a circular region of interest (4 mm in diameter) was positioned inside the image box, primarily in the outer renal cortex, to obtain the maximum elastic value displayed (Figure 1(A)). In a previous study, the maximum elastic value proved to be the best for distinguishing the extent of renal fibrosis when compared to other SWE parameter values [15]. Throughout the entire process, rigorous quality control measures were carried out, whereby measurements were deemed inadequate or failed in the case of weak or no signals in the elasticity image box [31]. For each subject, five independent and valid elastic values were obtained, and the arithmetical mean value was used in the statistical analysis.

### Renal biopsy

A US-guided percutaneous renal biopsy was performed in the right renal lower pole using an automatic 16- or 18-G needle. All specimens were embedded in paraffin and stained with hematoxylin-eosin, Masson’s trichrome, periodic acid-Schiff, and methenamine silver (Figure 1(B–E)). Then, the renal tissues were processed for light microscopy, immunofluorescence, and electron microscopy. Stained tissue sections were independently examined by two professional pathologists with 6–8 years’ experience in renal pathological diagnosis, who were blinded to clinical information and US features. In cases of disagreement, they reached a consensus after negotiation. A semi-quantitative system was used to assess the severity of renal fibrosis, as described in our previous research (Table 1) [15]. Based on pathological scores, patients were divided into three groups: mild fibrosis (9 points), moderate fibrosis (10–18 points), and severe fibrosis (19 points). Considering the small number of severe impairment cases (*n* = 18), we combined moderate and severe impairments as a moderate-severe category for comparison with mild impairment in the following analysis.

**Table 1.** Index of renal fibrosis pathology grade.

Score	Glomerular score (3–12 points)			Tubulointerstitial score (3–9 points)			Vascular score (2–6 points)	
	Glomerular hypercellularity	Glomerular segmental lesions	Glomerular sclerosis	Interstitial cell infiltration	Interstitial fibrosis	Tubular atrophy	Vessel wall thickening	Arterial hyaline change
1	<25%	<10%	<10%	<25%	<25%	<25%	<10%	<10%
2	≥25–50%	≥10–25%	≥10–25%	≥25–50%	≥25–50%	≥25–50%	≥10–25%	≥10–25%
3	≥50–75%	≥25–50%	≥25–50%	≥50%	≥50%	≥50%	≥25%	≥25%
4	≥75%	≥50%	≥50%	NA	NA	NA	NA	NA

NA: not applicable.

### Development and validation of the MLP model

The MLP classifier was used to construct the diagnostic model by fitting the SWE value, conventional US parameters, and readily accessible clinical features (including the aforementioned demographic information, liquid biopsy indicators, and comorbidity) in the training cohort. A grid search algorithm was employed to tune the hyperparameters of the classifier [32]. Due to the small sample size of this study, the entire training cohort was treated as a batch. During training, the model was optimized using the Vanilla Gradient Descent algorithm. As a measure of the discriminatory ability of the MLP model, the area under the curve (AUC) was calculated in accordance with the receiver operating characteristic (ROC) curve. The optimal cutoff point was determined by the Youden index method, and the corresponding sensitivity, specificity, and accuracy were calculated. The water-fall plot was employed to visualize the individual’s accuracy on the task. Using 1000 bootstrap re-samplings, a calibration curve was plotted to analyze the agreement between the observed and predicted results. Generally, the closer the correction line was to the diagonal, the better the prediction [33]. The goodness of fit was assessed with the Hosmer-Lemeshow test, and a non-significant *P* value (>0.05) indicated the model was well fitted. An evaluation of the clinical utility of the MLP model was conducted using decision curve analysis (DCA) in order to quantify the net benefits at different threshold probabilities [34]. Also, a clinical impact curve was plotted to determine the ratio of false-positive vs. true-positive values at different risk probability levels [35]. Discrimination and calibration, as well as clinical utility, were first tested in the training cohort and then validated in the test cohort. Moreover, we also constructed a logistic regression model by combining all the input variables for comparison with the MLP model in terms of discrimination capability.

### Statistical analysis

Statistical analyses were performed with SPSS 26.0 (IBM Corp. Released 2019. IBM SPSS Statistics for Windows, Version 26.0. Armonk, NY: IBM Corp) and Software R (version 4.1.2). To compare categorical variables presented as frequencies (percentages), a Chi-squared or Fisher’s exact test was used, while a Student’s *t*-test or Mann–Whitney *U*-test was used to compare continuous variables presented as means ± standard deviations (SD), or medians (interquartile ranges), as appropriate. As an indication of statistical significance, a two-sided *P* value of < 0.05 was considered.

## Results

### Characteristics of study cohort

The baseline characteristics of the training and test cohorts are presented in Table 2. With the exception of renal parenchyma thickness, demographic information, liquid biopsy indicators, comorbidities, and US parameters did not differ significantly. A total of 64 (56.14%) and 24 (50.00%) cases of moderate-severe renal fibrosis were found in the training and test cohorts, respectively, and no significant difference was observed between them (*p* = 0.474). The etiology of CKD is presented in Table S1.

**Table 2.** Demographic and clinical characteristics of patients with CKD in the training and test sets.

Characteristic	Training cohort (*n* = 114)	Test cohort (*n* = 48)	*P*-value
Demographic information			
Age (years)	41.05 ± 14.28	38.88 ± 14.26	0.377
Sex			0.990
Male	64 (56.14)	27 (56.25)	
Female	50 (43.86)	21 (43.75)	
BMI (kg/m^2^)	24.20 ± 3.60	23.94 ± 3.98	0.689
Liquid biopsy indicator			
eGFR (mL/min/1.73 m^2^)	81.92 ± 35.98	84.72 ± 35.09	0.650
Blood urea nitrogen (mmol/L)	5.37 (4.30–7.21)	5.57 (4.44–7.64)	0.408
Serum creatinine (umol/L)	87.00 (63.50–127.75)	85.00 (63.25–107.50)	0.685
Serum uric acid (umol/L)	388.54 ± 89.45	404.98 ± 119.41	0.337
Serum albumin (g/L)	33.60 ± 9.16	31.27 ± 10.05	0.153
UACR (g/gCr)	1.08 (0.23-2.37)	1.25 (0.19–3.47)	0.618
Ultrasound parameter			
Renal longitudinal diameter (cm)	10.35 ± 0.89	10.59 ± 0.87	0.121
Renal parenchyma thickness (cm)	1.57 ± 0.27	1.69 ± 0.29	0.017
RI	0.64 ± 0.07	0.63 ± 0.06	0.513
SWE value (kPa)	34.12 ± 10.37	34.99 ± 8.78	0.608
Comorbidity			
Diabetes			0.465
No	100 (87.72)	44 (91.67)	
Yes	14 (12.28)	4 (8.33)	
Hypertension			0.532
No	75 (65.79)	34 (70.83)	
Yes	39 (34.21)	14 (29.17)	
Cardiovascular disease			0.928
No	104 (91.23)	44 (91.67)	
Yes	10 (8.77)	4 (8.33)	
Severity of renal pathology			
Mild	50 (43.86)	24 (50.00)	0.474
Moderate-severe	64 (56.14)	24 (50.00)	

Categorical variables are presented as *n* (%) and continuous variables as mean ± standard deviation or median (interquartile range) as appropriate.

BMI: body mass index; Egfr: estimated glomerular filtration rate; UACR: urine albumin to creatinine ratio; RI: resistance index; SWE: shear wave elastography.

**Table 3.** Diagnostic performance of the MLP model.

Index	Training cohort				Test cohort			
	AUC (95% CI)	Sensitivity (95% CI)	Specificity (95% CI)	Accuracy (95% CI)	AUC (95% CI)	Sensitivity (95% CI)	Specificity (95% CI)	Accuracy (95% CI)
MLP model	0.93 (0.88–0.98)	0.89 (0.79–0.95)	0.88 (0.76–0.95)	0.88 (0.81–0.93)	0.86 (0.75–0.97)	0.79 (0.58–0.93)	0.83 (0.63–0.95)	0.81 (0.67–0.91)

MLP: multilayer perceptron; AUC: area under the curve; CI: confidence level.

### Construction of MLP model

**Figure 2.**
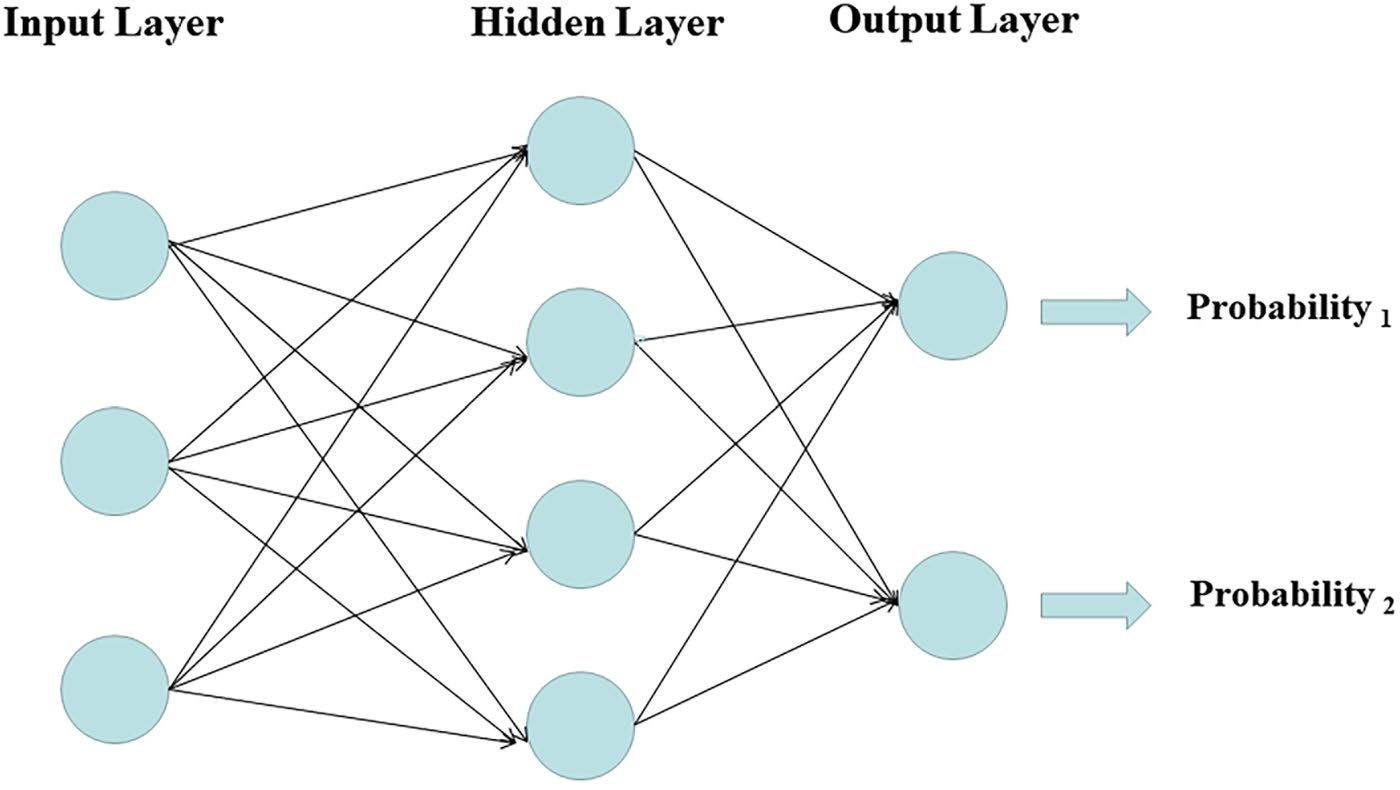
The established multilayer perceptron model consists of an input layer, a hidden layer, and an output layer.

**Figure 3.**
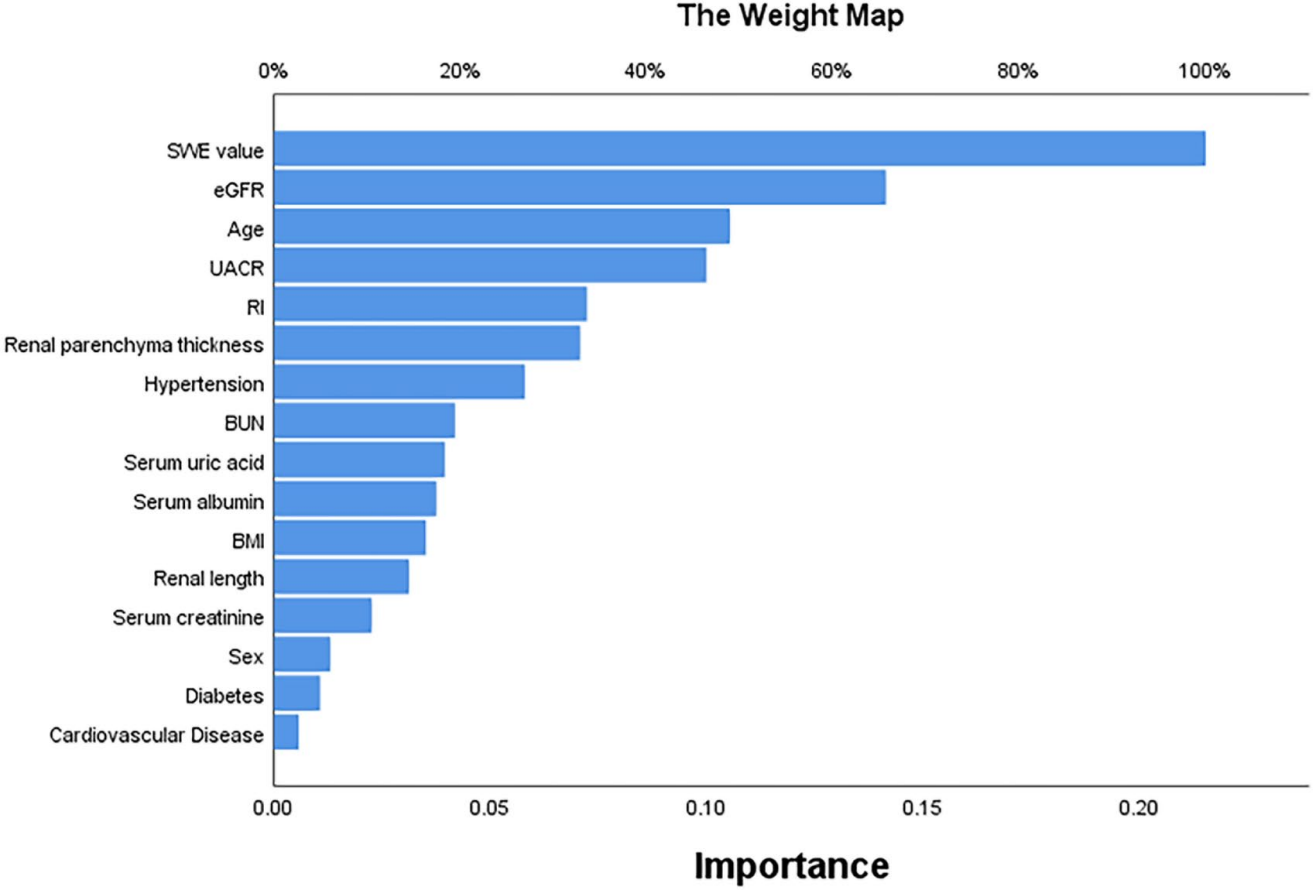
The relative importance of each predictor variable in the multilayer perceptron model. The longer the bar is represented by the variable, the greater the relative contribution of the variable to the model. SWE: shear wave elastography; eGFR: estimated glomerular filtration rate; UACR: urinary albumin creatinine ratio; RI: resistive index; BUN: blood urea nitrogen; BMI: body mass index.

A diagnostic model based on MLP classifier was developed by integrating 2D-SWE data with conventional US parameters as well as readily available clinical information, including demographics and liquid biopsy indicators (Figure 2). The architecture of the MLP model consists of an input layer, a hidden layer, and an output layer. The number of inputs and outputs is twenty and two, respectively, and the hidden layer contains four hidden units. Since the input layer does not involve calculation, the number of layers in the MLP model is two. The hidden layer is between the input layer and the output layer. The neurons in the hidden layer are fully connected to each input in the input layer, and the neurons in the output layer are fully connected to each neuron in the hidden layer. Thus, both the hidden layer and the output layer are fully connected. The activation functions of the hidden layer and output layer are hyperbolic tangent and softmax, respectively. The detailed network information of the MLP model is shown in Table S2. The relative importance of each variable in terms of outcome prediction ability is depicted in Figure 3, with 2D-SWE ranking as the top contributor, followed by eGFR, age, then UACR, and renal RI.

### Performance of MLP model

**Figure 4.**
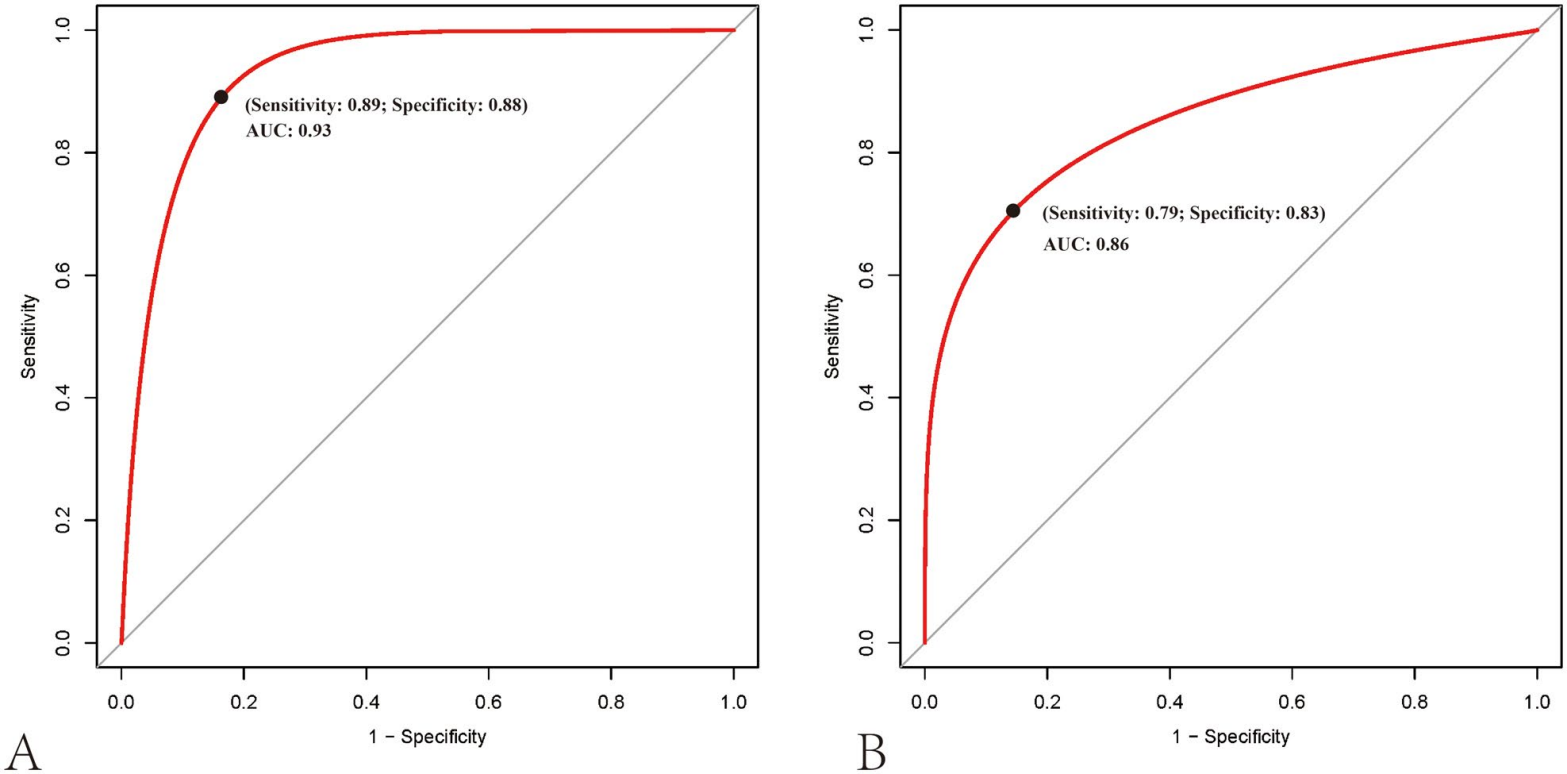
Receiver operating characteristic curves for differentiating moderate-severe renal fibrosis from mild one in the training (A) and test cohorts (B).

**Figure 5.**
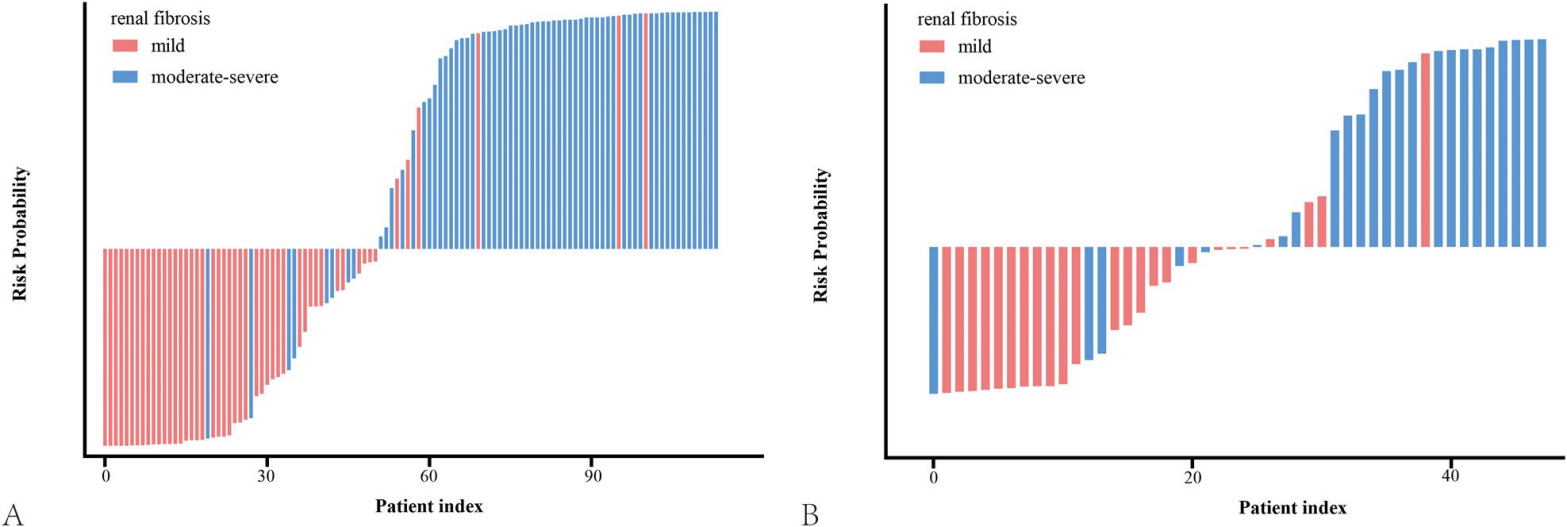
Water-fall plots constructed by the multilayer perceptron model in the training (A) and test cohorts (B). The blue area below the threshold indicates individuals with moderate-severe impairment who were misclassified as having mild impairment. The pink part above the threshold indicates individuals with mild impairment who were misclassified as moderate-severe impairment.

In the training set, the MLP model achieved satisfactory diagnostic performance, with an AUC of 0.93 [95% confidence interval (CI) = 0.88 to 0.98], the sensitivity of 0.89 [95% CI = 0.79 to 0.95], specificity of 0.88 [95% CI = 0.76 to 0.95], and accuracy of 0.88 [95% CI = 0.81 to 0.93] (Figure 4(A), Table 3). Also, the MLP model remained excellent in terms of diagnostic ability in the test set, yielding an AUC of 0.86 [95% CI = 0.75 to 0.97], a sensitivity of 0.79 [95% CI = 0.58 to 0.93], specificity of 0.83 [95% CI = 0.63 to 0.95], and accuracy of 0.81 [95% CI = 0.67 to 0.91] (Figure 4(B), Table 3), which was superior to the logistic model [test cohort: AUC = 0.79, 95% CI = 0.66 to 0.92; sensitivity = 0.70, 95% CI = 0.49 to 0.87; specificity = 0.83, 95% CI = 0.63 to 0.95]. To get a better understanding of how well the model can tell the difference between patients with moderate-severe fibrosis and those with mild fibrosis, a water-fall plot was created to visualize the diagnosis accuracy (Figure 5). Mild and moderate-severe renal fibrosis are depicted by pink and blue bars, respectively, while misclassified data is represented by blue and pink regions below and above the threshold. As shown in the water-fall diagram, the established MLP model has a high level of classification accuracy at the patient level.

**Figure 6.**
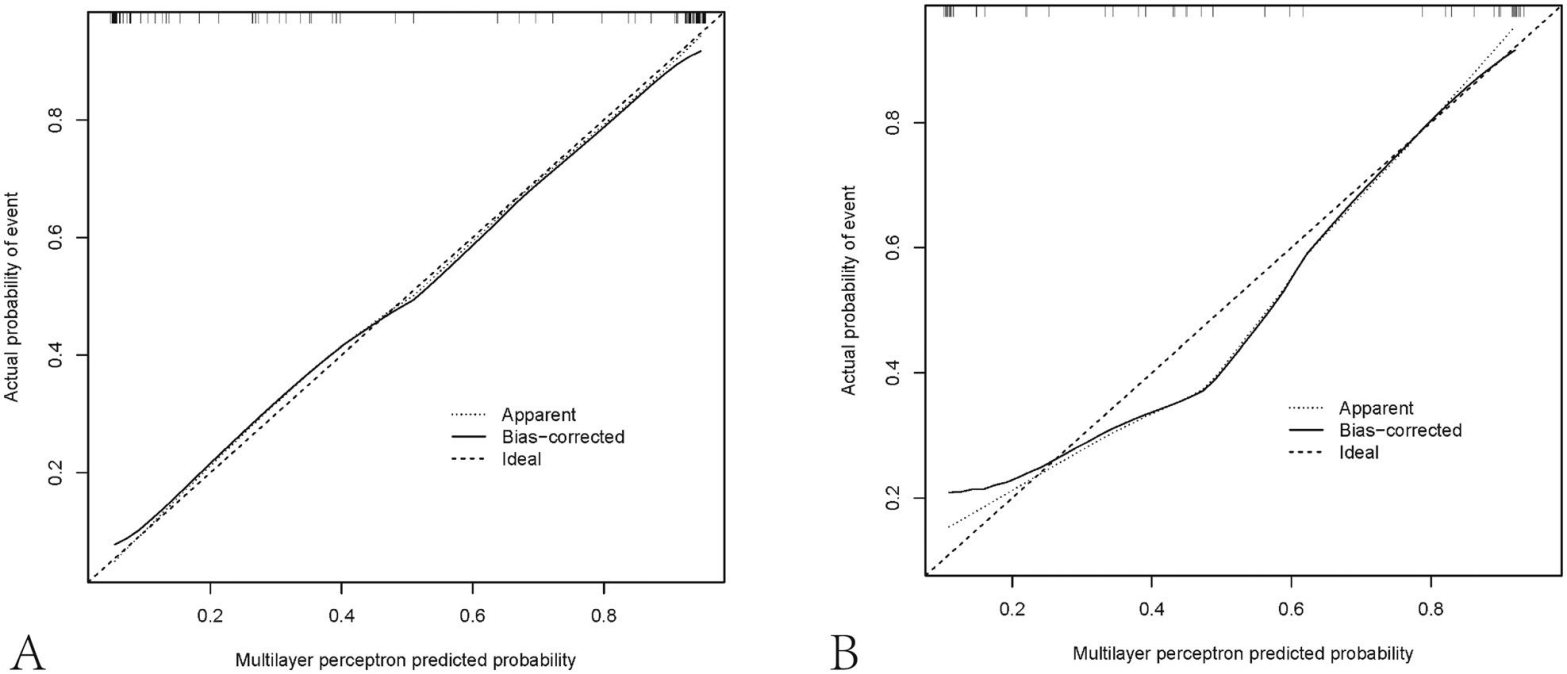
Calibration curves of the multilayer perceptron model prediction in the training (A) and test cohorts (B). Calibration curves depict the calibration of the established model in terms of the agreement between the predicted risks of moderate-severe renal pathological impairment and the observed outcomes of moderate-severe impairment. The y-axis shows actual moderate-severe impairment diagnoses, and the x-axis indicates the predicted moderate-severe impairment risk. The diagonal dotted line represents a perfect prediction by an ideal model. The solid line represents the performance of the model; a closer fit to the diagonal dotted line represents a more accurate prediction.

In the training and test cohorts, the Hosmer-Lemeshow test indicated a *P* value of 0.342 and 0.438, respectively, indicating that the model is well fitted. The calibration curves revealed good consistency with the real condition of the MLP model for the assessment of renal fibrosis in patients with CKD in the training and test sets (Figure 6).

### Clinical utility of MLP model

**Figure 7.**
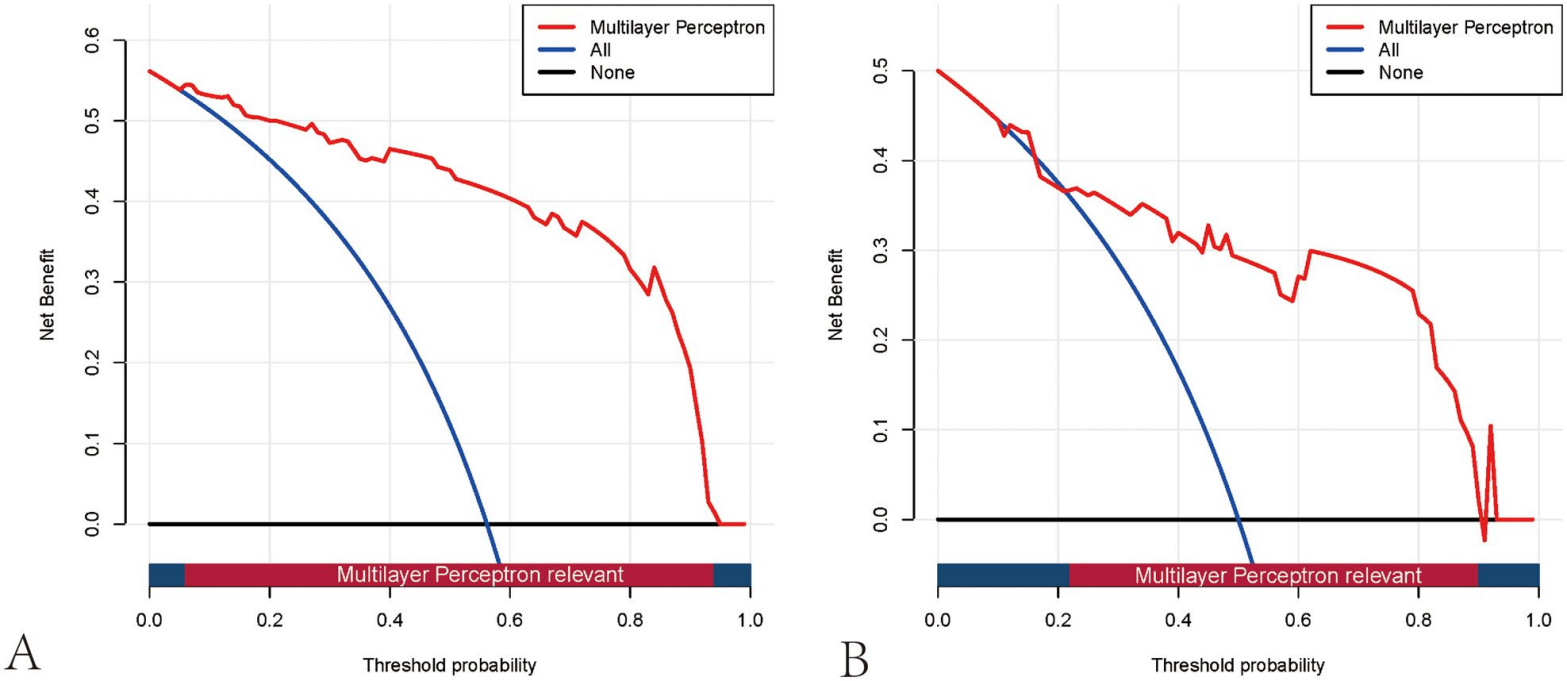
Decision curve analysis curves for the multilayer perceptron model in the training (A) and test cohorts (B). The y-axis shows the net benefit, and the x-axis indicates the risk threshold. The red line represents the prediction model. The blue line represents the assumption that all patients have moderate-severe renal pathological impairment. The black line depicts the assumption that none of the patients suffer from moderate-severe impairment. The net benefit was calculated by subtracting the proportion of false-positive patients from the proportion of true-positive patients, weighted by the relative harm of forgoing treatment compared with the negative consequences of unnecessary treatment.

**Figure 8.**
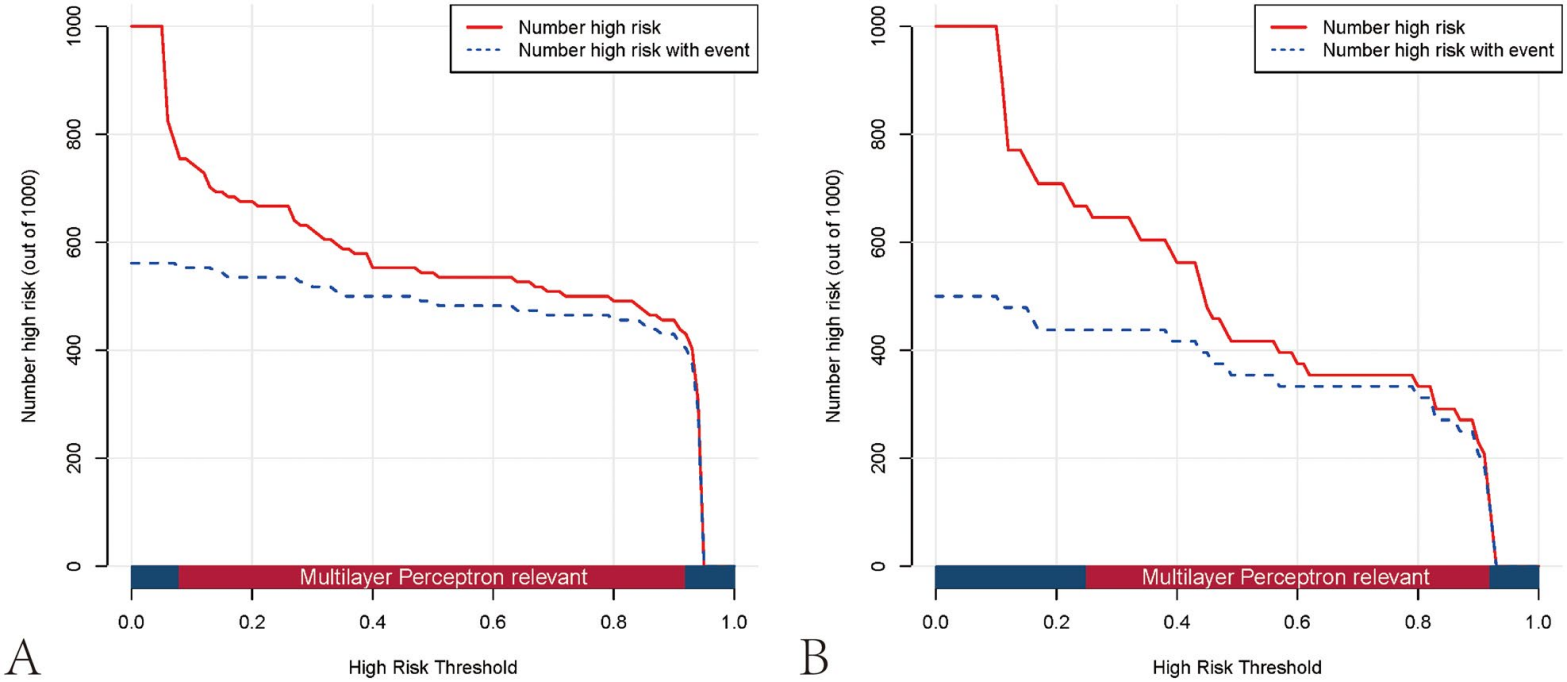
Clinical impact curves of the multilayer perceptron model in the training (A) and test cohorts (B). The y-axis measures the number of individuals at high risk, and the x-axis measures the risk threshold. The red curve shows how many out of 1000 patients the prediction model classifies as positive (high-risk) at each probability threshold. In contrast, the blue curve shows the number of true positives at each probability threshold.

Based on the DCA curve, the MLP model outperformed both the treat-none and treat-all strategies across a wide range of risk threshold probabilities, suggesting that the model demonstrates good clinical utility (Figure 7). In addition, the clinical impact curve was further plotted to intuitively appraise the potential clinical impact of using the MLP model for the evaluation of renal fibrosis in patients with CKD (Figure 8). When the risk threshold was >8% in the training cohort or >25% in the test cohort, the ratio of false-positive value to true-positive value was lower than 50%. Both the DCA curve and clinical impact curve indicate that the MLP model confers high practical value with low potential negative implications.

## Discussion

A MLP model was developed in the present study based on 2D-SWE and easily accessible clinical features for noninvasive assessment of renal fibrosis in patients with CKD and further validated. This established model had favourable diagnostic performance, fine calibration, and satisfactory clinical value. To the best of our knowledge, this is the first study to apply the MLP network classifier integrating 2D-SWE to CKD patients. The presented MLP models could be an adjuvant decision-support system for medical treatment decision-making as well as routine follow-up assessment.

As CKD progresses, different interventions for the various stages of renal fibrosis should be implemented to prevent exacerbation and improve prognosis [36–38]. Timely identification of individuals with mild renal fibrosis would enable them to avoid factors that contribute to or worsen the condition. Preventive measures could then be taken, thus delaying the progression of the disease. When treating CKD patients with moderate-severe renal fibrosis, more radical and aggressive treatment regimens are necessary to prevent the development and occurrence of complications while also delaying the initiation of dialysis therapy, which ultimately enhances survival and quality of life. Also, the severity of renal fibrosis impairment required constant reevaluation during the therapeutic process, which allowed clinicians to adjust treatment options accordingly, ultimately optimizing clinical decisions so as to achieve the most appropriate treatment efficacy for each individual patient. Hence, as a non-invasive, reliable diagnostic tool, the MLP model developed in this study is of substantial clinical relevance to assist clinicians in the accurate, personalized and dynamic assessment of renal fibrosis impairment in CKD patients.

With an AUC of 0.93 in the training cohort and 0.86 in the test cohort, the constructed MLP model effectively differentiated patients with moderate-severe renal fibrosis from mild patients. This performance outperformed those obtained with the 2D-SWE modality alone [AUC = 0.76; 95% CI = 0.68 to 0.85] [15] or the logistic model [AUC = 0.79; 95% CI = 0.66 to 0.92]. Moreover, the DCA curve and clinical impact curve demonstrated that the MLP model had favourable clinical application along with relatively few negative effects across a wide range of threshold probabilities, which resulted in advantages in clinical practice and personalized treatment options. Using an end-to-end deep learning framework, the MLP classifier could automatically derive variable weights based on the relevance between the input variables and output variables, avoiding the potential for selection bias caused by manual screening variables. With one or more hidden layers, the classifier was able to produce a higher-level and more abstracted feature selection algorithm; through the supervised learning modality, the classifier could automatically adjust the iteration process in the optimization algorithm, thereby ensuring sufficient generalization ability to prevent overfitting [18, 21]. As of today, the MLP classifier is widely applied in medical analyses in a wide range of fields, such as disease diagnosis, prognosis, therapy development, and treatment evaluation. Using a US-based MLP model, Liang T et al. distinguished breast mucinous cancer and its subtypes from fibroadenoma with an AUC ranging from 0.88 to 0.92 [20]. Yu J et al. used clinical features to develop an MLP model that predicted the severity and progression of carotid atherosclerosis in asymptomatic patients [19]. This model yielded an AUC value of 0.77 [95% CI = 0.75 to 0.77]. Meng Y et al. developed an MLP model incorporating MRI radiomics features for the prediction of fibroblast activation protein expression in pancreatic ductal adenocarcinomas, and this model also performed well [39]. The results of these studies further support the potential of the MLP model in clinical settings.

Several classical ensemble machine learning models (such as eXtreme Gradient Boosting and random forest) were also applied to address relevant clinical issues [40]. In terms of this research topic, ensemble models were able to provide fine diagnostic performance in the training cohort, with AUC values ranging from 0.97 to 1.00 (Table S3, Figure S1). However, in the test cohort, they were less effective, yielding an AUC of 0.77 to 0.78. It is likely that this result is due to overfitting. While ensemble models have been extensively employed in clinical research, many studies still require feature filtering (i.e., feature reduction and selection) prior to modelling to avoid dimensional disasters, which result in the loss of pertinent information [41, 42]. Once a large number of feature inputs are involved, it is easy for a model to fall victim to the "curse of dimensions" and the overfitting phenomenon [43]. Compared to tree models, the MLP model exhibits superior nonlinearity capabilities [44]. By mapping features to a higher-dimensional feature space, the MLP has the capability of selecting features automatically rather than through manual screening, therefore possessing the competitive advantages of high model efficiency, being less laborious, and so forth. As mentioned above, the MLP model can assign the weights of features on its own to complete the whole training process, known as an "end-to-end" training procedure. End-to-end training on raw data without feature engineering may be more beneficial for this medical problem than ensemble models.

The diagnostic performance of the MLP model depends to the greatest extent upon the following features: first, the SWE value, followed by eGFR and age, then UACR, and finally renal RI. In SWE, tissue stiffness can be evaluated by examining the speed of propagation of shear waves generated as a result of deformation caused by acoustic radiation force impulses being applied to target tissue [45]. Previous studies had demonstrated the clinical application of SWE in the evaluation of renal fibrosis [15, 16, 46]. Interstitial fibrosis and glomerulosclerosis have a trend of increasing in the aging kidney, whereas eGFR decreases with advanced age [47]. Furthermore, eGFR is widely accepted and has been used in routine clinical practice to assess CKD progression [22]. Proteinuria may be caused by inflammatory cells infiltrating the renal interstitium and the tubule-interstitium being replaced by a fibrotic scar [48]. Finally, renal RI was characterized as an influential predictor regarding the progress and prognosis of CKD patients, regardless of urine protein and eGFR levels [49, 50].

The application of SWE for the evaluation of renal fibrosis has been the subject of previous investigations [51–54]. It should be noted, however, that the present study presents some novelties in comparison with earlier studies. The implementation of SWE in this study is based on real-time imaging of the Mach cone principle with increased precision in measurements [55]. In contrast to prior studies that frequently necessitated manual feature screening through feature engineering, the current study adopts an end-to-end approach, thus obviating the need for time-consuming feature screening while concurrently achieving remarkable diagnostic outcomes. Additionally, this study extends beyond a simple evaluation of diagnostic performance by also examining the model’s calibration and clinical utility. To provide a more comprehensive evaluation of the model, additional models were constructed and compared with the one developed in this study. This serves to provide a more robust assessment of the model’s strengths and limitations.

There are still some limitations associated with this study. First, the sample size of this study was relatively small, especially in severe cases of renal fibrosis, and a larger population should be included for analysis in the future. Second, this was a single-center study, which could be further validated *via* external datasets or multi-center settings for generalization. Third, radiomics is an emerging approach to medical imaging analysis that can provide additional clinically useful information. We will consider investigating this promising technique to solve the relevant medical issue in future studies.

## Conclusion

In our study, an MLP model based on 2D-SWE and clinical features was developed which had an excellent diagnostic performance as well as clinical utility for the noninvasive assessment of renal fibrosis in patients with CKD. This novel technique helps refine clinical decision-making and provides information about the disease’s risk. Despite some progress being made on the MLP model developed in the present study, the research results were derived from a single-center cohort with a small sample size, and future studies are therefore needed to validate these findings in a multi-center, large population-based cohort setting.

## Supplementary Material

Supplemental Material

## Data Availability

The data presented in this study are available from the corresponding author upon reasonable request. Data are not publicly available due to privacy or ethical concerns.
