# Supplementary material for: Using elastography-based multilayer perceptron model to evaluate renal fibrosis in chronic kidney disease
Source: Ren Fail. 2023 Apr 19;45(1):2202755. doi: 10.1080/0886022X.2023.2202755 (PMC10120461; doi:10.1080/0886022X.2023.2202755)
Supplement: Supplemental Material [file IRNF_A_2202755_SM3118.pdf]

Table S1. Etiology of CKD

Diagnosis	Number
IgA nephropathy	72 (44.4%)
Membranous nephropathy	34 (21.0%)
Minimal change nephropathy	16 (9.9%)
Mesangial proliferative glomerulonephritis	9 (5.6%)
Lupus nephritis	9 (5.6%)
Focal segmental glomerular sclerosis	8 (4.9%)
Diabetic nephropathy	6 (3.7%)
Others	5 (3.0%)
Unknowns	3 (1.9%)

Notes: Categorical variables are presented as n (%).

Abbreviations: CKD, chronic kidney disease.

Table S2. The Network Information of MLP model

Layer	Parameter	Variable
Input Layer	Factors	Sex
		Diabetes
		Hypertension
		Cardiovascular Disease
	Covariates	Age
		BMI
		eGFR
		BUN
		Serum creatinine
		Serum uric acid
		Serum albumin
		UACR
		Renal length
		Renal parenchyma thickness
		RI
		SWE value
	Number of Units[a]	20
	Rescaling Method for Covariates	Standardized

Hidden Layer(s)	Number of Hidden Layers	1
	Number of Units in Hidden Layer 1[a]	4
	Activation Function	Hyperbolic tangent
Output Layer	Dependent Variables	Moderate-severe renal fibrosis
	Number of Units	2
	Activation Function	SoftMax
	Error Function	Cross-entropy

Note: a. Excluding the bias unit.
Abbreviations: MLP, multilayer perceptron; BMI, body mass index; eGFR, estimated glomerular filtration rate; BUN, blood urea nitrogen; UACR, urinary albumin creatinine ratio; RI, resistive index; SWE, shear wave elastography.

Table S3. Diagnostic performance of the ensemble models

Index	Training cohort				Test cohort			
	AUC (95% CI)	Sensitivity (95% CI)	Specificity (95% CI)	Accuracy (95% CI)	AUC (95% CI)	Sensitivity (95% CI)	Specificity (95% CI)	Accuracy (95% CI)
XGBoost	0.97 (0.94-0.99)	0.94	0.93	0.92	0.77 (0.63-0.91)	0.71	0.88	0.71
Random Forest	1.00 (1.00-1.00)	1.00	1.00	0.98	0.78 (0.64-0.91)	0.71	0.83	0.73

Abbreviations: XGBoost, eXtreme Gradient Boosting; AUC, area under the curve; CI, confidence level.

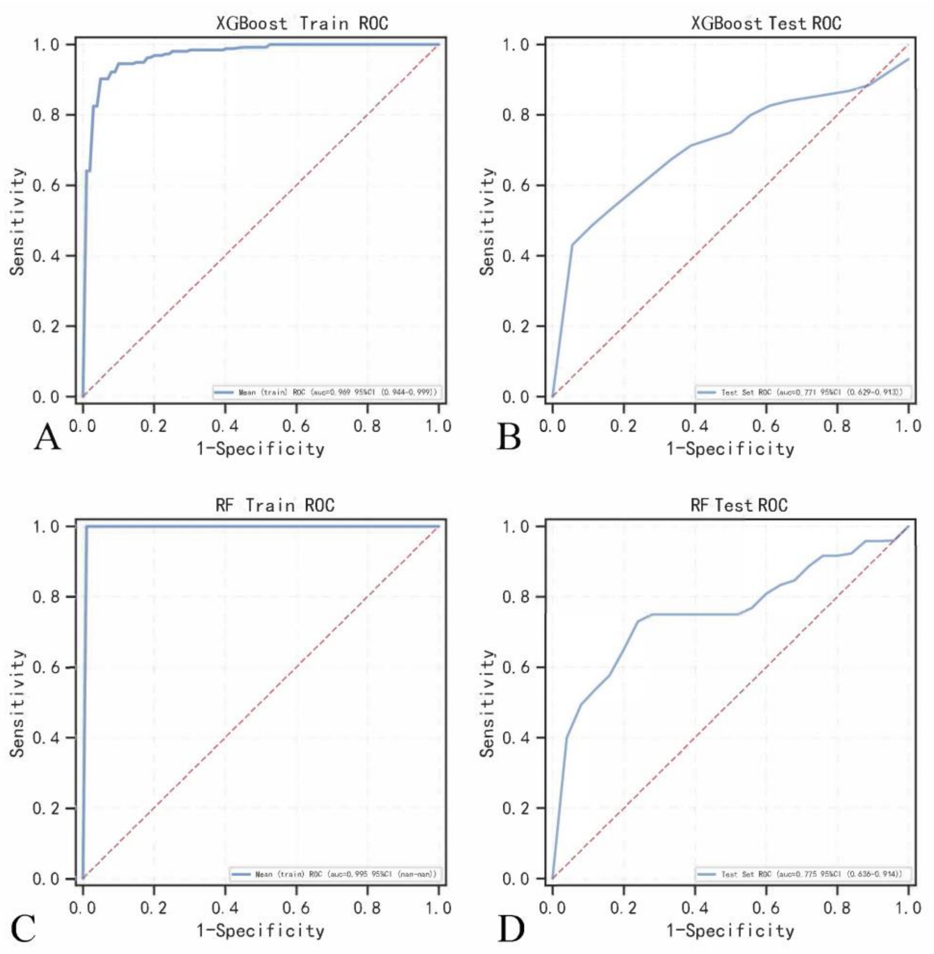

Figure S1. ROC curves of the ensemble models
